# Coeliac Disease, Microscopic Colitis, and Exclusion Diets—A Commemorative Issue in Honor of Dr. Fernando Fernández-Bañares

**DOI:** 10.3390/nu17091537

**Published:** 2025-04-30

**Authors:** Maria Esteve, Beatriz Arau, Albert Martín-Cardona

**Affiliations:** 1 Gastroenterology Department, Hospital Universitari Mútua Terrassa, University of Barcelona, 08221 Terrassa, Spain; 2 Centro de Investigación Biomédica en Red de Enfermedades Hepáticas y Digestivas (CIBERehd), 28029 Madrid, Spain

## 1. Introduction

On the occasion of the passing of Dr. Fernando Fernández-Bañares, a world-renowned researcher, several colleagues from the fields to which he devoted much of his life have paid tribute by contributing their recent research to this Special Issue of *Nutrients*. Celiac disease (CD) and associated disorders—such as microscopic colitis, eosinophilic esophagitis, and dermatitis herpetiformis—are the focus of the studies included in this issue, which are summarized below.

### 1.1. An Overview of Key Aspects of Celiac Disease Based on the Published Articles

Celiac disease has been identified in every country where it has been investigated. However, its prevalence and incidence vary widely across regions and over time. Most published studies and meta-analyses report an increasing number of diagnosed cases. One meta-analysis estimated a global seroprevalence of 1.4%, with 0.7% confirmed by biopsy [1]. This study, along with a more recent meta-analysis on CD incidence [2], demonstrated an upward trend, with higher rates observed in children compared to adults and in women compared to men.

A recent European meta-analysis focused on the pediatric population also supports this progressive increase [3]. However, the authors note significant limitations in the available studies, particularly in the use of heterogeneous methodologies, which account for the wide variation in reported prevalence (ranging from 0.10% to 3.03%).

Since CD more frequently affects women and children, failing to adjust prevalence estimates according to age and sex introduces significant biases that cannot be attributed solely to geography or temporal factors. Furthermore, most meta-analyses combine two main study types: (1) clinical records of diagnosed cases and (2) serological screenings of asymptomatic populations, the latter providing a more accurate estimate of the true prevalence. To determine whether CD prevalence is truly increasing, standardized methodologies and longitudinal studies in well-defined regions are essential.

A recent longitudinal study in genetically predisposed children showed high regional variability in cumulative incidence, highlighting the influence of environmental, genetic, and epigenetic factors—even within a single country [4]. Among these, viral infections [5] and changes in gut microbiota [6] have drawn particular research interest.

In this issue, we present an epidemiological study from Catalonia reporting a 50% reduction in pediatric CD prevalence compared to the previous decade. Both studies, conducted ten years apart, followed identical methodologies. The reduction was significantly associated with the widespread implementation of the rotavirus vaccine [7]. Thus, identifying both triggering and protective factors for CD should be a central goal of future research [8].

### 1.2. Diagnostic Challenges and Innovations

Another key research area in CD involves improving diagnostic methods in complex clinical situations. The introduction of specific serological tests at the end of the 20th century revolutionized CD diagnosis [9]. However, diagnostic challenges persist, particularly in the following scenarios: (1) seronegative villous atrophy (VA), (2) mild celiac enteropathy, and (3) patients already on a gluten-free diet (GFD) seeking diagnostic confirmation.

Several studies in this issue evaluate the diagnostic utility of the so-called celiac lymphogram using flow cytometry—especially the TCR-γδ+ intraepithelial lymphocytes (IELs). The celiac lymphogram is characterized by an increased percentage of TCR-γδ+ IELs and a decreased percentage of CD3− IELs [10].

Multiple studies report that seronegative VA accounts for 11–39% of CD cases, depending on the severity of the lesion. This proportion rises to about 90% in cases of mild enteropathy—also known as lymphocytic enteritis or duodenosis [11,12,13]. Although often referred to as “potential” CD [14], this mild gluten-related enteropathy may present with symptoms and laboratory abnormalities identical to those in cases of villous atrophy [15]. Therefore, it should be considered part of the CD spectrum, regardless of its histological mildness. This form accounts for about 15% of cases of lymphocytic enteritis [13], and the percentage increases among individuals with high-risk HLA genotypes. However, similar “sprue-like enteropathies” may be caused by other agents, such as *Helicobacter pylori*, NSAIDs, parasites, and others, making the differential diagnosis of seronegative CD particularly challenging [12,13].

The current criteria for diagnosing seronegative CD include the exclusion of all other causes of VA, the presence of CD-compatible HLA genotypes, and—when needed—a gluten challenge to re-induce the lesion [12]. A multicenter study of 67 patients with seronegative VA (37 with CD, 30 without) demonstrated that the celiac lymphogram has high diagnostic accuracy, with both sensitivity and specificity exceeding 90% [16].

Another difficult scenario is diagnosing CD in patients who have already started a GFD and experienced clinical improvement. Differentiating between CD and non-celiac gluten sensitivity (NCGS) is challenging, as most CD-related histological and immunological alterations are reversed after gluten withdrawal. Though a gluten challenge is the current gold standard [17], many patients are unwilling to undergo it. Moreover, the standard gluten challenge protocol often fails to reinduce detectable mucosal damage in a substantial number of patients.

In this issue, we present a study assessing the kinetics of TCR-γδ+ IELs after a long-term GFD. Although it was known that these cells may persist in the duodenum despite gluten withdrawal, their exact long-term progression and long-term evolution in NCGS patients were previously unknown. Our results show that most CD patients—both Marsh 3 and Marsh 1—retain elevated γδ+ T-cell levels after more than two years on a GFD, whereas most NCGS patients present with normal levels that decrease over time. Additionally, one-third of CD patients normalize CD3− IELs values after two years, while NCGS patients show erratic patterns. Thus, assessing γδ+ T cells alone may allow for a reliable differential diagnosis. A cut-off of TCR-γδ+ >13.3% yields a diagnostic accuracy of 0.88 for CD in patients already on a GFD [18].

Other proposed diagnostic methods for patients on a GFD include the following: (1) the detection of gut-homing CD8+ T cells in peripheral blood after a 3-day, 10 g/day gluten challenge [19]; (2) measurement of plasma IL-2 after a single gluten dose [20]; and (3) gluten-specific CD4+ T-cell analysis using HLA-DQ2 tetramers and IFN-γ ELISPOT assays [21,22]. Comparative clinical studies are needed to evaluate their utility alone or in combination.

### 1.3. Non-Responsive and Refractory CD

It is well established that GFD leads to both clinical and histological remission in most CD cases, reducing mortality and long-term complications. However, a subset of patients continues to experience symptoms or persistent duodenal lesions despite strict adherence. This condition, known as non-responsive CD, is defined as persistent symptoms, signs, or laboratory abnormalities despite a strict GFD [23]. It is a broad term encompassing refractory CD as well as other causes.

In cases where symptoms persist but histology is normalized, comorbidities—such as microscopic colitis [24], eosinophilic esophagitis, or irritable bowel syndrome—should be considered. IBS symptoms may also be exacerbated by dietary FODMAPs. In this issue, a systematic review demonstrates that low-FODMAP diets can benefit CD patients who do not fully respond to a GFD [25].

Another frequent issue is persistent villous atrophy, despite apparent clinical and serological responses. While gluten exposure is a common cause, it is not the only one. Studies show that persistent atrophy is more frequent in adults than in children, likely due to age-related immune changes or increased sensitivity to tracing gluten intake [26]. It is hoped that emerging pharmacological therapies will support mucosal healing in refractory or non-responsive patients [27].

CD is a promising target for drug development, due to its well-characterized pathogenesis. Therapeutic strategies under investigation include glutenases to degrade toxic peptides, anti-IL-15 antibodies, larazotide to enhance tight junctions, inhibitors of tissue transglutaminase-mediated deamidation, and agents that induce immune tolerance by suppressing gluten-specific CD4+ T cells.

### 1.4. An Overview of the Published Articles

The diverse aspects of CD research highlighted in the editorial are thoroughly addressed in this monographic issue. The study by Arau et al. (contribution 2) illustrates that CD does not follow a uniform global upward trend. It underscores the influence of environmental factors on disease prevalence and emphasizes that comparisons over time require identical methodological approaches to be valid. Diagnostic strategies represent a major focus of this issue. One particularly relevant line of research is the development of point-of-care serological tests, which enable immediate case detection and thus contribute to reducing both underdiagnosis and diagnostic delay. In this context, the study by Mendia I et al. (contribution 1) confirms the accuracy of a rapid test based on anti-tTG-IgA antibodies. Among the contributions on diagnostic tools, several studies provide new insights into the clinical application of the celiac lymphogram, particularly the assessment of TCR-γδ+ IELs (contributions 3, 5, 6, and 10). As noted in the editorial, the utility of this biomarker in the diagnostic workup of complex cases is well established. González-Castro, A.M. et al. (contribution 9) present a narrative review on the association between microscopic colitis (MC) and CD, which coexist in approximately 6% of patients. As highlighted, this association should be considered especially in those who show an incomplete response to a GFD. In such cases, screening for MC in patients with CD—and vice versa—is recommended. This clinical message is further supported by the study by Raju, S.A. (contribution 7), which explores the clinical characteristics of hospitalized patients with MC and the implications of a concomitant CD diagnosis.

The coexistence of diet-related disorders increases the complexity of dietary management. The relationship between CD and eosinophilic esophagitis (EoE) remains under investigation, although recent population-based data indicate a six-fold increase in the risk of CD among patients with EoE. The study by Arial, Á et al. (contribution 8) demonstrates that dietary therapy continues to be effective for EoE, particularly in pediatric populations and in cases involving multiple food eliminations. As previously discussed, the systematic review by Lusetti, F et al. (contribution 4) confirms the benefit of a low-FODMAP diet in CD patients with partial or absent responses to gluten withdrawal. Nevertheless, the gluten-free diet remains the cornerstone of CD management.

Fiora, F et al. (contribution 11) compare the results of two surveys conducted ten years apart, assessing patient perspectives on the GFD in terms of adherence, perceived limitations, and concerns. Despite generally good adherence, the authors underscore the need for international guidelines to standardize both diagnostic and follow-up processes, including access to expert dietary counseling. In line with this, the study by Crespo-Escobar, P et al. (contribution 12) highlights the importance of improving nutritional education among both patients and healthcare providers.

## 2. Conclusions

In summary, this Special Issue dedicated to Dr. Fernando Fernández-Bañares addresses key current and future research directions in CD and associated disorders. Scientific progress results from the efforts of researchers working collaboratively across borders. This monographic issue, featuring contributions from different countries, exemplifies such collaborative work aimed at improving human health and well-being.
